# At-home infectious disease testing: An idea whose time has come

**DOI:** 10.1017/ash.2022.307

**Published:** 2022-10-13

**Authors:** Amesh A. Adalja

**Affiliations:** Johns Hopkins Center for Health Security, Bloomberg School of Public Health, Baltimore, Maryland

*“Nothing is more powerful than an idea whose time has come.” – Victor Hugo*

A hallmark of the coronavirus disease 2019 (COVID-19) pandemic in its current stage is the ubiquity of home diagnostic testing for severe acute respiratory coronavirus virus 2 (SARS-CoV-2). These tests have been touted for their varied uses to facilitate navigating a world in which SARS-CoV-2 is an ever-present consideration. Apart from HIV at-home testing, COVID-19 is one of the only conditions that can be tested for at home with an almost immediate result. At-home infectious diseases testing has gained significant momentum during the COVID-19 pandemic given the myriad cascading positive benefits the technology offers. Currently, plans for home influenza and RSV tests are underway, as well as tests for sexually transmitted infections (STIs). These efforts began before the pandemic and have only accelerated as COVID-19 home tests have demonstrated their value. Here, I discuss how at-home infectious disease tests can be harnessed to optimize individual treatment outcomes and positively influence public health efforts. I have explored many of these themes in a prepandemic report,^1^ and the ensuing years have concretized many of the theoretic benefits.

## The clinical benefits of at-home testing for infectious disease

The fundamental question asked by a patient to a clinician is often, “What am I sick with?” With infectious diseases, a diagnostic test is often relied upon to provide an answer and to initiate appropriate management. When a patient is given a diagnosis, it leads to several follow-up actions, all of which apply whether the diagnosis is obtained at home or in a traditional medical setting.

### Linkage to effective treatment

The most obvious follow-up action of a diagnosis for an infectious disease is for treatment, if available. For example, a positive COVID-19 home test in a high-risk symptomatic patient often leads to interaction with a physician who will first assess a patient’s symptoms and risk for progression to severe illness, which may prompt a prescription for nirmatrelvir-ritonavir or an appointment for an infusion of a therapeutic antiviral agent. Similarly, a positive home test for HIV almost always leads to interaction with a physician and linkage to care. For HIV, such testing could be game changing because earlier treatment significantly diminishes immunological damage, contagiousness, and future comorbidities.^2^ More prompt treatment could have similar benefits with COVID-19 and its long-term sequelae. Much of this can be greatly enhanced with the use of telemedicine platforms that have been widely adopted during the pandemic.

Such benefits are far-reaching, beyond COVID-19 and HIV. For example, consider influenza. The rate of antiviral prescribing for influenza is largely suboptimal,^3^ and this has many deleterious effects on the burden of seasonal influenza including longer duration of illness, more severe illness, and increased rates of healthcare utilization. Home influenza tests that use molecular diagnostics, such as loop-mediated isothermal amplification (LAMP), have been under development for several years, as well as those employing improved rapid antigen-detection technology (which have been deployed in clinical settings).^4,5^

Influenza antivirals diminish the duration of symptoms in ordinary cases of influenza. Importantly, these medications can be lifesaving in severe cases of influenza or in the treatment of patients at high risk for severe outcomes. However, many patients with influenza are not prescribed antivirals due to the lack of a specific diagnosis, a diagnosis made too late for maximal antiviral benefit, the use of historically poorly sensitive influenza diagnostic tests, or lack of awareness. Those with influenza who are not treated with antivirals are more likely to suffer severe consequences such as pneumonia, respiratory failure, and death.^3^ These issues will become more salient if the influenza antiviral oseltamivir reaches planned over-the-counter status^6^ and with single-dose antivirals such as baloxavir (oral) peramivir (intravenous infusion).

Likewise, if home STI testing comes to fruition, treatment of patients and their sexual partners for gonorrhea, chlamydia, and other infections could be greatly expedited. As became evident during the COVID-19 pandemic, the lack of access to testing for common STIs played a role in their increased rates during the pandemic.^7^ Likewise, the potential benefits of at-home testing for monkeypox are also being recognized. The diagnosis and appropriate treatment of group A streptococcal pharyngitis could also be improved in a similar fashion, which would be of great utility in pediatrics to rapidly identify and treat cases and reduce secondary spread among family members and classmates.

### Diminishing inappropriate treatments for antimicrobial stewardship

Many individuals with viral upper respiratory infections are inappropriately prescribed antibacterial agents. This poor practice pattern is abetted when there is no specific diagnosis given for a patient’s symptoms. With a positive test for a respiratory virus, clinicians are less likely to prescribe an ineffective or inappropriate antibiotic, and the patient will be free from exposure to potential side effects while also diminishing *Clostridioides difficile* infection risk, preserving the gut microbiome, and reducing antibiotic resistance.^8^

### Behavioral changes

On an individual level, understanding COVID-19, influenza, or other respiratory virus positivity status can be tremendously empowering in terms of determining personal behaviors such as school or work attendance and isolation from household contacts. A positive test result for a communicable infectious disease may also prompt an individual to evaluate their behaviors to decrease risk of transmitting illness to others. This phenomenon was seen with HIV home testing^9^ and has been a mainstay of the advocacy of home COVID-19 testing being considered as a contagiousness test.^10^ This could also combat the healthcare industry culture of presenteeism in which workers may show up sick and contagious.^11^

## The public health benefit of at-home testing for infectious disease

Despite some lamentations during the COVID-19 pandemic that the advent of home testing diminished the situational awareness of public health authorities and rendered certain metrics such as daily case counts and percent test positivity unreliable, the benefits incalculably outweighed those concerns. The tests, as one advocate often put it, “put the public in public health” because of the actions some individuals took to isolate more rapidly and notify close contacts. Indeed, many public health departments had no way to assimilate home test information, which will be a crucial barrier to overcome to maximize the societal benefit of this approach.

With home influenza, HIV, or STI tests, the same public health benefit will accrue and, over time, will diminish the societal impact of infection and increase public health and patient-centered benefits such as less waiting room or school transmission of influenza, for example.

Also, as mentioned earlier, less inappropriate antibiotic use and more optimal antiviral use will have positive public health benefits by decreasing rates of drug resistance and reducing severe disease that may prompt hospitalization.

## Barriers to widespread at-home infectious diseases testing

Although there is great potential for at-home diagnostic testing for infectious disease, important challenges must be resolved to fully realize the potential of these paradigm-shifting technologies.

Test manufacturers should partner with telemedicine platforms and primary care physicians who have televisit capacity to optimize linkage to care and medical treatment. Put simply, the following actions are necessary for optimal delivery of care: (1) Symptom onset prompts a home test. (2)If test is positive, package instructions link to consultation with a telemedicine provider, or the patient can consult their own primary care physician (ie, in person, by telephone, or by video visit). And (3)provider conducts risk assessment and prescription or appointment for antiviral infusion as appropriate. This type of provider-access model would improve uptake and add value; however, linkage to relevant public health authorities is a key challenge. Although anonymity and patient privacy concerns are important in HIV home testing, the use of these tests for respiratory viruses may face less scrutiny. However, as these tests reach commercial markets, developing technological solutions to allow test results to be transmitted to local health departments, state health departments, or the CDC will be crucial, and this capacity is currently lacking with COVID-19 home tests. Such data may need to be anonymized to the ZIP code (or partial ZIP code) level, but these data can still be useful in elucidating the burden of infection in each area and in detecting unusual clusters of positive results.

Another factor in the success of these technologies will be the degree of public uptake. The public may be reticent to incur out-of-pocket costs for a test which, if negative may not directly link to any ameliorating therapy. Determining the price point at which uptake will be maximized is an important task for manufacturers. If insurers and other third-party payers, including the Centers for Medicare and Medicaid (CMS), could be persuaded of the benefits of home testing as means of curbing healthcare costs, it may have a substantial impact on uptake.

Lastly, although these tests may primarily target consumers, this does not preclude their use in medical practices and congregate settings like nursing facilities, schools and colleges, etc, which may increase uptake in the market.

## The disruptive vision of success

In the future, I envision a world in which at-home infectious diseases testing is routinized and employed for unequivocal benefits the patient, the clinician, and public health agencies. This future is not far off; the technology already exists and could be realized within 5 years.

A clear linkage with public health information systems is a priority; however, the information gained remains extremely useful even if not all positive results are reported into a database. Tests could include QR codes or Bluetooth technology for voluntary reporting. Public health agencies could partner with a sample of users to gain situational awareness of the incidence of specific infections for which home testing is widely available. Moreover, tests coupled with telemedicine provider visits could be directly reported by clinicians to relevant public health authorities.

Clinical Laboratory Improvement Amendments (CLIA)–waived technology already exists for easy-to-use multiplex panels that individuals could purchase and use at home. An aspirational vision of success is for such devices to be networked and linked to provide (1) a diagnosis to the user, (2) access to a provider for possible treatment, and (3) information to public health agencies. The true potential of such devices could be harnessed in a community outbreak setting as part of mitigation strategies, or to provide early signals that a novel pathogen may be present if multiple individuals with respiratory symptoms test negative for the usual battery of viruses (smart thermometers can provide a similar but less-refined warning^12^). Additionally, if individuals had such devices at home, public health authorities could distribute new cartridges to users to test for novel pathogens in the event of an outbreak. Myriad potential public health benefits will result from better management of outbreaks of both routine and novel respiratory pathogens with expanded home testing. Such a scenario is not currently being pursued or evaluated, but it is a paradigm to advocate.

Health authorities could also deploy such tests to sentinel populations to monitor disease activity among these groups. Akin to distributing HIV testing kits to individuals at high risk for HIV acquisition, agricultural workers who have high-risk contact with animals, frequent travelers, nursing home residents, and other groups could be monitored more closely with such tests with close follow-up of positive results.

Although antiviral medications provide a major incentive for influenza and COVID-19 diagnostic testing, they do not exist for most other respiratory viruses. Attention given to COVID-19 and influenza testing can spread to other diagnostics for viruses that are typically neglected in formal surveillance activities, such as respiratory syncytial virus, which is not a uniformly reportable condition in all states. By understanding the burden of these other viruses, an incentive for the development of antivirals and vaccines targeting these other pathogens may develop.

Barriers to this future state include costs, public uptake, regulatory agency buy-in—all of which are surmountable. As medicine moves into the 21st century and amazing technology has become ubiquitous in most homes, the advancement of medical diagnostics for infectious diseases in the home setting is overdue.
